# Optimisation of PET framing sequences

**DOI:** 10.1186/2197-7364-1-S1-A60

**Published:** 2014-07-29

**Authors:** Miguel Patrício, Liliana Caldeira

**Affiliations:** Laboratory of Biostatistics and Medical Informatics, IBILI – Faculty of Medicine, University of Coimbra, Kragujevac, Portugal; Institute of Neurosciences and Medicine at the Forschungszentrum Jülich, Kragujevac, Germany

Our goal is to propose and evaluate a method for choosing framing sequences for Positron Emission Tomography (PET) acquisition protocols. Data detected by PET scanners is translated into concentration of radiotracer by using image reconstruction methods. Most of these methods require a sequence of frame durations to be defined *a priori*. After reconstruction, the PET data needs to be quantified and combined with MR images to allow statistical intersubject comparisons. The resulting kinetic parameters then depend on the framing sequence adopted. However, though the literature offers a set of such sequences to choose from for common radiotracers, there are no methods for optimising this choice.

We generated perfect reference and tissue data with the simplified reference tissue method (SRTM) [1] model, using data from the literature for [11C]-Raclopride and by choosing parameter values for regions of high binding and low binding (R_1_=1.15, k_2_=6.3E-3, BP=3.3 and R_1_=1.15, k_2_=6.3E-3, BP=4.3E-1, respectively), [2]. A list-mode data file simulating dynamic PET data for the Siemens 3T MR-BrainPET was further generated, using the Utah phantom. It was such that the time activity curves obtained after reconstruction correspond to the perfect curves. The total number of true counts was set to 100 million.

Statistical error was then added to the list-mode file data, which was subsequently reconstructed using the Ordinary Poisson Ordered Subset Expectation Maximisation (OP-OSEM) method in PRESTO and adopting different framing sequences. After reconstruction and quantification using a basis function implementation of SRTM [3], the resulting kinetic parameters were compared to the exact solution, cf. [4]. By comparing the errors obtained for the binding potential, at each voxel, using the framing sequences proposed in [1] and [3], we show that clear differences arise when using different framing sequences to compute kinetic parameters.
